# Chytrid fungus infection in zebrafish demonstrates that the pathogen can parasitize non-amphibian vertebrate hosts

**DOI:** 10.1038/ncomms15048

**Published:** 2017-04-20

**Authors:** Nicole Liew, Maria J. Mazon Moya, Claudia J. Wierzbicki, Michael Hollinshead, Michael J. Dillon, Christopher R. Thornton, Amy Ellison, Jo Cable, Matthew C. Fisher, Serge Mostowy

**Affiliations:** 1 Section of Microbiology, MRC Centre of Molecular Bacteriology and Infection (CMBI), Imperial College London, London SW7 2AZ, UK; 2 Department of Infectious Disease Epidemiology, Imperial College London, London W2 1PG, UK; 3 Division of Virology, Department of Pathology, Cambridge University, Cambridge CB2 1QP, UK; 4 College of Life and Environmental Sciences, Geoffrey Pope Building, University of Exeter, Exeter EX4 4QD, UK; 5 School of Biosciences, Cardiff University, Cardiff CF10 3AX, UK

## Abstract

Aquatic chytrid fungi threaten amphibian biodiversity worldwide owing to their ability to rapidly expand their geographical distributions and to infect a wide range of hosts. Combating this risk requires an understanding of chytrid host range to identify potential reservoirs of infection and to safeguard uninfected regions through enhanced biosecurity. Here we extend our knowledge on the host range of the chytrid *Batrachochytrium dendrobatidis* by demonstrating infection of a non-amphibian vertebrate host, the zebrafish. We observe dose-dependent mortality and show that chytrid can infect and proliferate on zebrafish tissue. We also show that infection phenotypes (fin erosion, cell apoptosis and muscle degeneration) are direct symptoms of infection. Successful infection is dependent on disrupting the zebrafish microbiome, highlighting that, as is widely found in amphibians, commensal bacteria confer protection against this pathogen. Collectively, our findings greatly expand the limited tool kit available to study pathogenesis and host response to chytrid infection.

## Abstract (web summary)

New infections of the amphibian chytrid fungus could arise from other animal reservoirs in the environment. Here, Liew *et al*. demonstrate that zebrafish can be infected by chytrid similarly to amphibians, expanding our understanding of how this pathogen can parasitize its hosts.

Pathogens that exhibit a broad host range constitute a growing threat to biodiversity. This owes to their intrinsic ability to undergo range expansions through human-mediated long-distance dispersal, then spill over and cause subsequent infection of naive hosts^1^,^2^. Pathogenic fungi exhibit the broadest spectrum of host ranges known for any group of pathogens, with aquatic chytrid fungi in the genus *Batrachochytrium* emerging as an extinction threat to amphibian species worldwide^1^,^3^. Two species of amphibian parasitizing chytrids have thus-far been described, *Batrachochytrium dendrobatidis* (*Bd*) and *Batrachochytrium salamandrivorans* (*Bsal*); of these *Bd* is known to parasitize all three orders of amphibians with nearly 700 species of amphibian infected to date^4^. Infection by *Bsal* thus far appears to be limited to caudates (salamanders) with fewer than 20 species known to be infected^5^.

The mechanisms underlying the ultra-generalist nature of *Bd* compared to its more host-restricted sister species have not yet been described. However, they likely include the ability to deeply invade and establish within the epidermal cells of the stratum corneum through lytic processes and to modulate host immunity through secreted factors^6^,^7^. While *Bd* infection was originally assumed to be restricted to amphibians, other non-amphibian hosts are thought to sustain infections. Key to this argument are studies showing that crayfish tissues are parasitized by *Bd* in nature, that these infections cause crayfish mortality, and that the infection could be transmitted to co-housed amphibians^8^. The nematode *Caenorhabditis elegans* is thought to also be parasitized by *Bd* with attendant mortality, and the keratin-rich toenails of waterbirds may act as a transient substrate for *Bd*^9^,^10^. However, more recent research finds no evidence for roles of non-vertebrate hosts in sustaining infections^11^. An unexamined factor that may confound all of these studies is whether commensal bacteria either interact synergistically with *Bd* to exacerbate infection through dysbiosis, or rather protect against infection through the production of antimicrobial compounds^12^. A microbial role in defining host range is likely to be important as an increasing number of studies are now showing associations between bacterial communities and *Bd* infections of amphibians in the field^13^,^14^,^15^. Taken together, these studies are important as they show that we are missing significant aspects of the biological interactions that define the host range of *Bd*.

A thus-far unexamined aspect of *Bd*'s epidemiology is its potential ability to infect other aquatic vertebrates, principally freshwater fish. If *Bd* were able to successfully parasitize fish, then this would represent an unparalleled opportunity for studying infection through use of the widely available zebrafish (*Danio rerio*). Recent studies have uncovered host immune responses of *Bd* infection in amphibians, though most of this work focused on adaptive immunity despite research suggesting an important role of the innate immune system, especially at the early stages of infection^16^,^17^,^18^,^19^,^20^,^21^. The fully developed innate immune system present in zebrafish larvae, along with its natural transparency, make it possible to easily study host-pathogen interactions in real time using non-invasive live-cell microscopy^22^,^23^,^24^,^25^. Furthermore, the immediate environment of the zebrafish larvae can easily be manipulated with antibiotics, allowing the role of commensal bacteria in disease dynamics to be studied. Thus, a zebrafish larvae infection model would present a novel opportunity to study pathogenesis and innate immune responses during *Bd* infection *in vivo.*

Here we tested whether zebrafish larvae can act as carriers of *Bd* by developing a 3-day dose-dependent model in which infection can be detected and quantified. We show that different stages of *Bd* infection can be observed on zebrafish larvae using histopathology and confocal microscopy, alongside symptoms of infection that are comparable to those observed in amphibians. These results show that zebrafish larvae undergo an infection process similar to that of *Bd*'s native amphibian hosts, highlighting the utility of zebrafish larvae as an important model to study this emerging panzootic infectious disease.

## Results

### *Bd* infection of zebrafish larvae

**Figure 1:**
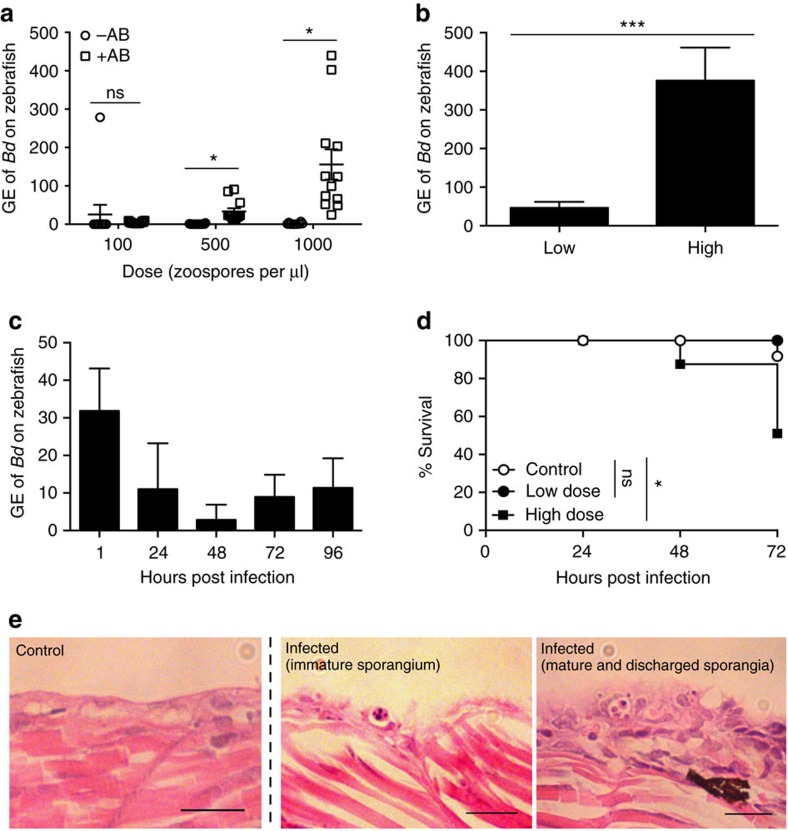
*Bd* infection of zebrafish larvae. (**a**) Zebrafish larvae bath water without (−AB, open circles) or with (+AB, open squares) 1% penicillin/streptomycin was inoculated with three doses of *Bd* zoospores. Bath water changed at 24 h post infection (h.p.i.) and larvae incubated for 72 h.p.i. Zebrafish DNA was extracted and amplified by qPCR. Data from 1 experiment shown, using *n*=12 per treatment. Mean±s.e.m. are shown. Significance testing performed using unpaired Student's *t*-test (two tailed), ns *P*>0.05, *P<0.05. See Supplementary Fig. 1b for replicate experiments. (**b**) Zebrafish larvae bath water was inoculated with low (<200 zsp per μl) or high (>200 zsp per μl) dose *Bd* zoospores and incubated for 72 h.p.i. Zebrafish DNA was extracted and amplified by qPCR. Data from two experiments plotted here, using *n*=12 per treatment. Mean±s.e.m. are shown. Significance testing performed using unpaired Student's *t*-test (two tailed), ***P<0.001. See Supplementary Fig. 1c for replicate experiments. (**c**) Zebrafish larvae bath water was inoculated with low dose *Bd* zoospores and incubated for 1, 24, 48, 72 or 96 h.p.i. Zebrafish DNA was extracted as in **a**; Data from one experiment are plotted here (dose=80 zsp per μl), using *n*=3 per time point. Mean±s.e.m. are shown. See Supplementary Fig. 1d for replicate experiments. (**d**) Survival curve of zebrafish larvae bath water inoculated with mTGhL (control, open circles, *n*=420), low (filled circles, *n*=84) or high (filled squares, *n*=336) dose *Bd* zoospores. Larvae were incubated for 72 h.p.i. Data pooled from twenty-five experiments. Mean±s.e.m. are shown. Significance testing performed by Mantel–Cox (log-rank) test. ns *P>*0.05, *P<0.05. See Supplementary Table 1 for replicate experiments. (**e**) Zebrafish larvae bath water was inoculated with control or high dose *Bd* zoospores and incubated for 72 h.p.i. Representative longitudinal histological sections shown here. 1, encysted zoosporangium; 2, mature sporangium with internal zoospores; 3, empty zoosporangium with discharge tube protruding out of epithelial cells exhibiting extensive hyperplasia. Scale bars, 20 μm. GE, genomic equivalents.

To investigate if zebrafish larvae at three days post fertilization (d.p.f.) can be infected with *Bd* zoospores (that is, the infectious stage of *Bd*), a bath water infection model was developed (Supplementary Fig. 1a). To investigate infection burden, we quantified the amount of *Bd* DNA on infected larvae using quantitative PCR (qPCR), an assay typically used to detect *Bd* infection *in vivo* using swabs or toe clips^18^,^26^. Initial experiments clearly showed an important role of commensal bacteria in mitigating against *Bd*'s ability to infect larvae. In the presence of antibiotics, intensities of infection were significantly greater when compared to larvae without antibiotic treatment (Fig. 1a, Supplementary Fig. 1b). Subsequently, all further experiments on zebrafish larvae were carried out in the presence of antibiotics. Samples tested show significantly higher genomic equivalents (GE) of *Bd* DNA on *Bd*-infected larvae compared to their bath water alone. We observed significantly higher GE of *Bd* DNA on larvae infected with >200 zoospores per μl (zsp per μl; herein referred to as high dose) of *Bd* zoospores when compared to those infected with <200 zsp per μl (herein referred to as low dose) of *Bd* zoospores, suggesting a dose-dependent nature of infection (Fig. 1b, Supplementary Fig. 1c). We performed qPCR time-course studies and detected *Bd* DNA on larvae up to 96 h post infection (h.p.i.; Fig. 1c; Supplementary Fig. 1d). To test whether we could extend our findings to include other species of fish, juvenile ornamental guppies (*Poecilia reticulata)* were infected with *Bd* zoospores. A qPCR time-course study performed on these guppies showed significantly higher GE of *Bd* DNA on guppies infected with live zoospores compared to those infected with the same dose of heat-killed zoospores at 5 days post infection (d.p.i.; Supplementary Fig. 1e). However, the GE of *Bd* DNA in live zoospore treated guppies became increasingly similar to control values at later time points (12 and 19 d.p.i., respectively), suggesting that infection is unable to persist in this host.

We observed that zebrafish larvae infected with a low dose of *Bd* zoospores show 100% survival, whereas larvae infected with a high dose of *Bd* zoospores show significant mortality from 0 to 72 h.p.i., supporting that infection is dose dependent (Fig. 1d; Supplementary Table 1). To visualize *Bd* infection in larvae we performed histopathology using methods commonly employed to confirm infection in amphibians^27^. We observed different life-history stages of *Bd* on infected larvae, including mature zoosporangia (that is, the reproductive stage of *Bd*) and empty discharged sporangia with a discharge tube protruding out of infected tissue. These images prove that *Bd* is able to both encyst and proliferate within the skin of zebrafish larvae (Fig. 1e), a finding that is consistent with the increased detection of GE of *Bd* by qPCR on infected larvae up to 96 h.p.i. The morphology of these *Bd* life-history stages are consistent with those found widely on *Bd-*infected amphibians, such as *Xenopus laevis*^7^,^28^. Taken together, these results show that zebrafish larvae can be infected with *Bd* in the presence of broad spectrum antibiotics, and that *Bd* infection of larvae resembles that of amphibians.

### Symptoms of *Bd* infection in zebrafish larvae

**Figure 2:**
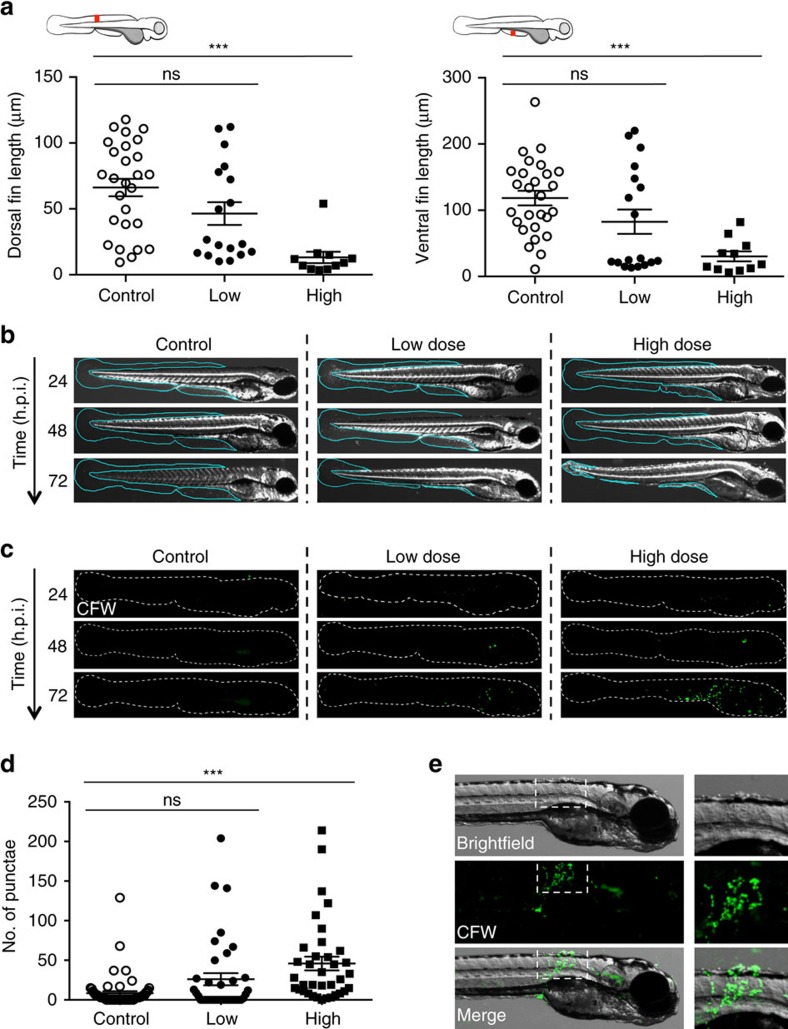
Symptoms of *Bd* infection in zebrafish larvae. (**a**) Zebrafish larvae bath water was inoculated with mTGhL plate washings (control, open circles), low (<200 zsp per μl, filled circles) or high (>200 zsp per μl, filled squares) dose *Bd* zoospores. Larvae were incubated for 72 h.p.i., where dorsal and ventral fin length was measured (as depicted by red line in cartoon above each graph). Each point represents an individual larva. Data pooled from three experiments per dose, using *n*=4–12 per treatment. Mean±s.e.m. are shown. Significance testing performed using unpaired Student's *t*-test (two-tailed), ns *P>*0.05, ***P<0.001. (**b**) Zebrafish larvae bath water was inoculated with control, low or high dose *Bd* zoospores, incubated for 72 h.p.i. and imaged by stereomicroscopy. Representative images with cyan outline showing presence or erosion of fin over time. (**c**) Zebrafish larvae bath water was inoculated with control, low or high dose *Bd* zoospores and incubated for 24, 48 or 72 h.p.i., then labelled with calcofluor white (CFW; for chitin, green) and imaged by fluorescent stereomicroscopy. Representative images with dotted outline of larvae showing (CFW)-labelled punctae on infected larvae. (**d**) Enumeration of CFW-labelled punctae from zebrafish larvae whose bath water was inoculated with control (open circles), low (filled circles) or high (filled squares) dose *Bd* zoospores and incubated for 72 h.p.i. Each point represents an individual larva. Data pooled from three experiments per dose, using *n*=12 per treatment. Mean±s.e.m. are shown. Significance testing performed using Mann–Whitney test (two tailed), ns *P>*0.05, ***P<0.001. See also Supplementary Fig. 2e for replicate experiments. (**e**) Zebrafish larvae bath water was inoculated with high dose *Bd* zoospores and incubated for 72 h.p.i., then labelled with CFW (green) and imaged by fluorescent stereomicroscopy. Representative images with insets showing colocalization of CFW-labelled punctae with blisters.

*Bd* infection in a wide range of amphibians can be accompanied by hyperplasia, hyperkeratosis of the skin, lethargy and loss of righting reflex^29^. Infected larvae showed erosion of tail fin, disruption of smooth muscle striations and blistering of skin at 72 h.p.i. (Supplementary Fig. 2a). To test whether these phenotypes are dose dependent, fin erosion was quantified by measuring two different widths of zebrafish larva caudal tail fin at 72 h.p.i. (Fig. 2a). When compared against control larvae, low or high dose infected larvae showed a 1.7±0.3- or 7.3±3.5-fold decrease in dorsal fin length, and a 1.6±0.1- or 7.1±2.7-fold decrease in ventral fin length, respectively. To investigate the onset of these infection phenotypes, time-course imaging experiments were performed and showed that morphological changes appeared between 48 and 72 h.p.i. (Fig. 2b). To test whether these phenotypes were caused by a factor associated with increased organic matter or zoospore secreted toxins in *Bd*-infected water, we performed time-course imaging studies with exposure of larvae to heat-killed *Bd* zoospores or *Bd* zoospore supernatant. Both treatments showed fin erosion similar to control larvae at all time points tested (Supplementary Fig. 2b,c). These results suggest that fin erosion is largely a consequence of *Bd* infection, although the precise roles of pathogen invasion and/or release of cytolytic factors from adherent *Bd* in establishing this phenotype remains to be determined.

A hallmark of chytrid infection in amphibians includes the presence of zoosporangia and rhizoid-like structures in the keratinized epidermal tissue layers of host skin^28^,^30^. To investigate symptoms of zebrafish larvae infection, *Bd*-infected larvae were labelled with calcofluor white (CFW). We first observed that CFW clearly labelled the chitinous cell wall of *Bd* sporangia in broth culture (Supplementary Fig. 2d). At 72 h.p.i., infected larvae showed fluorescent punctae (bright round spots) throughout the entire body and fin (Fig. 2c). Time-course imaging experiments showed that these phenotypes appeared within 48–72 h.p.i. (Fig. 2c), consistent with the appearance of fin erosion (Supplementary Fig. 2c). We quantified the number of CFW-positive punctae on each larva and observed that larvae infected with a high dose of *Bd* zoospores show significantly more (4.7±0.6-fold) CFW-positive punctae compared to controls (Fig. 2d, Supplementary Fig. 2e). Larvae infected with a low dose of *Bd* zoospores show similar values to controls, highlighting the dose dependent nature of infection. Moreover, CFW-positive punctae colocalized with blister-like structures on eroded muscle of infected larvae (Fig. 2e), suggesting that both CFW-positive punctae and blistering of skin are symptoms of infection by *Bd*.

### Consequence of *Bd* infection on zebrafish larvae host tissue

**Figure 3:**
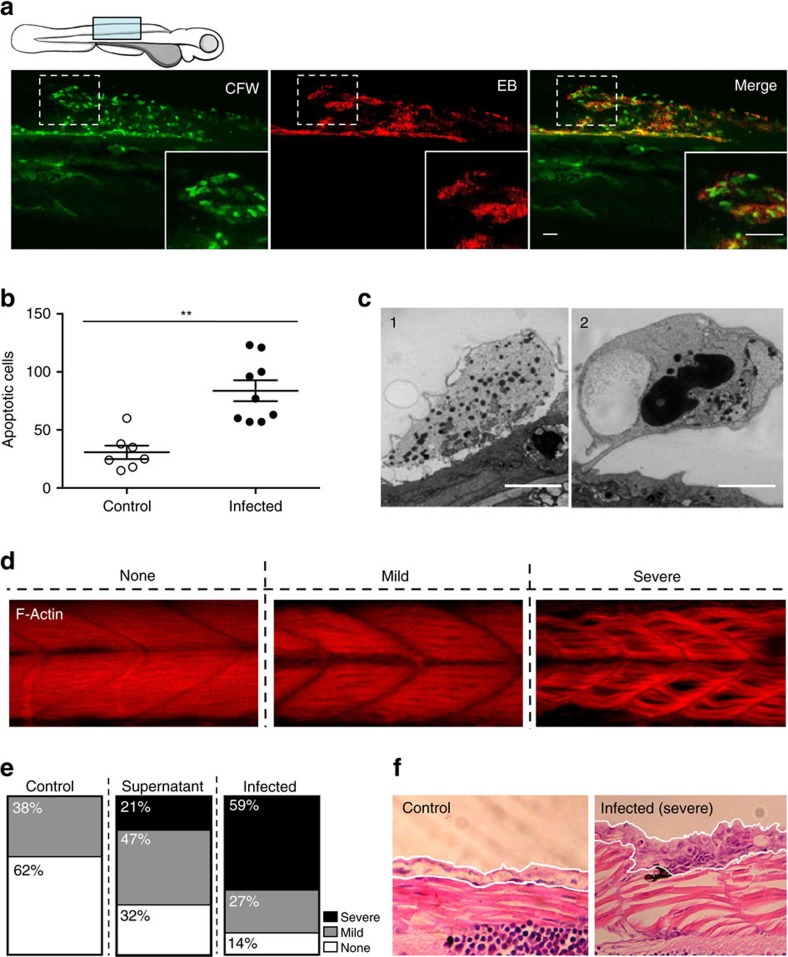
Consequence of *Bd* infection on zebrafish larvae host tissue. (**a**) Zebrafish larvae bath water was inoculated with low (<200 zsp per μl) dose *Bd* zoospores and incubated for 72 h.p.i., then labelled with calcoluor white (CFW; for chitin, green) and evans blue (EB; for tissue damage, red), and visualized by confocal microscopy. Images taken at × 40, maximum intensity projection of *Z*-stack shown here. Cartoon depicts imaged region. Representative images with insets highlight colocalization of CFW-labelled punctae with EB positive tissue damage. Scale bars, 50 μm. See also Supplementary Fig. 3a for more examples. (**b**) Enumeration of apoptotic cells from larvae whose bath water was inoculated with mTGhL plate washings (control, open circles) or low (filled circles) dose *Bd* zoospores and incubated for 72 h.p.i., then fixed and labelled with TUNEL. Each point represents an individual larva. Data pooled from three experiments, using *n*=3 per treatment. Mean±s.e.m. are shown. Significance testing performed using Mann–Whitney test (two tailed), **P<0.01. (**c**) Zebrafish larvae bath water was inoculated with high dose *Bd* zoospores and incubated for 72 h.p.i., then fixed for electron microscopy (EM). 1, Sloughing necrotic cell. 2, sloughed necrotic cell. Scale bars, 1 μm. See also Supplementary Fig. 3d for EM images of larvae treated with control. (**d**) Zebrafish larvae bath water was inoculated with control, high (>200 zsp per μl) dose *Bd* zoospore supernatant or high dose *Bd* zoospores and incubated for 72 h.p.i., then fixed, labelled with phalloidin (for F-Actin; red) and visualized by confocal microscopy. Images taken at 40X, maximum intensity projection of *Z*-stack shown here. Representative images of larva with no, mild or severe muscle degeneration are shown here. (**e**) Proportion of larvae with no (white), mild (grey) or severe (black) muscle degeneration when prepared as in **d**. (**f**) Zebrafish larvae bath water was inoculated with control or high dose *Bd* zoospores and incubated for 72 h.p.i. Representative longitudinal histological sections shown here. White outline highlights skin of larvae, which in infected image shows hyperplasia of epithelial cells with mature and discharged sporangia.

We used confocal microscopy to investigate the consequence of *Bd* infection on zebrafish larvae. *Bd*-infected larvae were fluorescently labelled with CFW and Evans Blue (EB) to detect tissue damage. CFW-positive punctae were observed on the fins and muscle of low dose infected larvae, which colocalize with EB-positive tissue damage (Fig. 3a, Supplementary Fig. 3a). Moreover, both CFW-positive punctae and EB-positive tissue damage were found to colocalize with fin erosion and blister-like structures on *Bd*-infected larvae (Supplementary Fig. 3b).

Recent studies have shown that exposure to *Bd* results in apoptosis of amphibian cells *in vitro*^6^,^31^. To test whether *Bd*-infected zebrafish larvae show a similar phenotype *in vivo*, TUNEL staining for apoptotic cells was performed. Infected larvae showed a 3.0±0.4-fold increase in number of TUNEL-positive apoptotic cells compared to controls (Fig. 3b). Apoptotic cells were mostly observed around fin edges and on the surface of larvae (Supplementary Fig. 3c), thus were likely to be epithelial cells where keratin is highly expressed^32^. Transmission electron microscopy showed necrotic epithelial cells sloughing off in infected larvae, as compared to healthy skin cells in control larvae (Fig. 3c; Supplementary Fig. 3d). Together, these results strongly suggest that *Bd* is damaging host tissue during infection of zebrafish larvae.

By 72 h.p.i., infected larvae showed disruption of clear muscle striations (Supplementary Fig. 2a). To further investigate this phenotype, larvae were fluorescently labelled with phalloidin to visualize actin filaments *in vivo*. Infected larvae showed striking muscle degeneration (Fig. 3d). Severity of muscle degeneration was classified into three categories based on the number of key phenotypes larval muscle fibres possessed, including loose packing, thinning, crumpling and deep tissue degeneration (Supplementary Fig. 3e). Using this classification system, high-dose-infected larvae show a higher percentage of severe muscle degeneration compared to *Bd* zoospore supernatant treated larvae, and both of these treatments show a higher percentage of severe muscle degeneration compared to controls (Fig. 3e). These results indicate that muscle degeneration may, at least in part, be the result of toxins secreted by *Bd* following infection. Histopathology on infected larvae also showed hyperplasia of skin where sporangia have formed, adjacent to severe muscle degeneration (Fig. 3f). These results demonstrate that *Bd* infection in zebrafish larvae is underpinned by severe tissue damage caused by secreted toxins and the infection process.

### Intracellular colonization by *Bd* in zebrafish larvae

**Figure 4:**
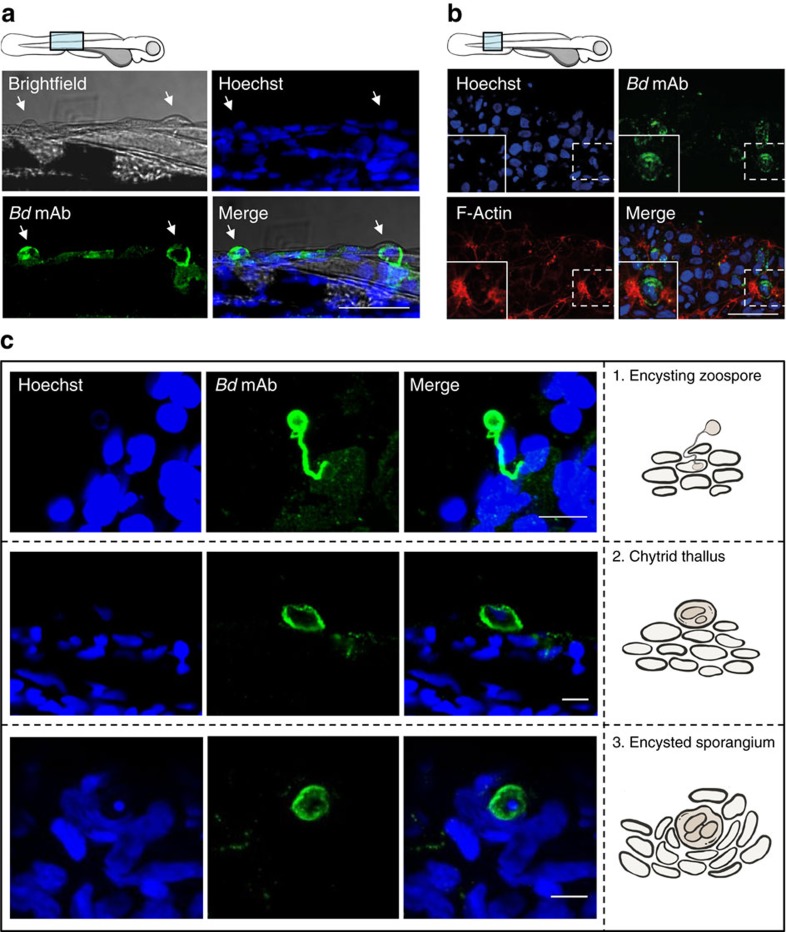
Intracellular colonization by *Bd* in zebrafish larvae. (**a**) Zebrafish larvae bath water was inoculated with high (>200 zsp per μl) dose *Bd* zoospores and incubated for 72 h.p.i., then fixed and labelled with Hoechst (for DNA; blue) and mAb 5C4 (for *Bd*; green) for visualization by confocal microscopy. Images taken at 63 X, maximum intensity projection of *Z*-stack shown here. Cartoon depicts imaged region. Representative images with arrows highlight colocalization between *Bd* and blisters on larvae skin. Scale bar, 50 μm. (**b**) Zebrafish larvae bath water was inoculated with high dose *Bd* zoospores and incubated for 72 h.p.i., then fixed and labelled with Hoechst (for DNA; blue), mAb 5C4 (for *Bd*; green) and phalloidin (for F-Actin; red) for visualization by confocal microscopy. Images taken at × 63, maximum intensity projection of *Z*-stack shown here. Cartoon depicts imaged region. Representative images with insets highlight *Bd* adjacent to host cell actin rearrangements. Scale bar, 50 μm. See also Supplementary Fig. 4c for host cell actin in control treated larvae. (**c**) Zebrafish larvae bath water was treated as in **a**. Images taken at × 40 or × 63, maximum intensity projection of *Z*-stack shown here. Images showing different stages of *Bd* invasion and infection on larvae, also depicted using cartoons in the right column. 1, rhizoid-like germ tube attached to encysting zoospore, 2, chytrid thallus growth on zebrafish larvae skin, 3, encysted sporangium amongst hyperplasic epithelial cells. Scale bars, 10 μm.

To determine how symptoms of infection in zebrafish larvae are related to the colonization of *Bd* on larvae skin, we labelled infected larvae at 72 h.p.i. with a novel *Bd-*specific monoclonal antibody, mAb 5C4. This antibody binds to a carbohydrate epitope on an extracellular antigen produced by *Bd*^33^, labelling both *Bd* zoospores and zoosporangia with minimal background labelling *in vivo* (Supplementary Fig. 4a,b). Infected larvae present areas of skin harbouring the *Bd* secreted antigen (Fig. 4a). Antibody labelling also colocalizes with blisters and actin reorganization on infected larvae, showing disruption of host tissue in response to *Bd* infection (Fig. 4a,b, Supplementary Fig. 4c). These results are consistent with the pattern of CFW-labelled punctae described above, indicating that mAb 5C4 is suitable for highly specific, in-depth analysis of the *Bd* infection process. Indeed, various stages of *Bd* infection can be visualized in infected larvae by 72 h.p.i. (Fig. 4c), including germ-tubes invading epidermal cells, encysting zoospores and intracellular zoosporangia amongst hyperplastic epithelial cell build-ups. These results show that zebrafish larvae undergo an infection process similar to that of amphibians, and that *Bd* infection of zebrafish can be powerful model system to study the invasion and proliferation of *Bd in vivo.*

## Discussion

Our study shows that *Bd* is able to infect and multiply on zebrafish larvae treated with antibiotics in a dose dependent manner that mimics the process of infection seen in amphibians. This demonstration of a non-amphibian vertebrate host being infected by *Bd* widens the host range previously known to be exploited by this hypervirulent chytrid lineage. Using a *Bd* monoclonal antibody (mAb 5C4), we were able to image the different stages of *Bd* infection with unprecedented resolution *in vivo*. Collectively, these results validate zebrafish larvae as a powerful aquatic model system within which these host-*Bd* interactions can be more fully explored. Furthermore, our observations that treating zebrafish with antibiotics results in higher burdens of infection highlight the use of probiotic bacteria to combat *Bd* infection^34^.

Although the specific host factors necessary for *Bd* infection remain to be discovered, *Bd* is commonly found to parasitize the keratinized tissue of both amphibian and non-amphibian hosts^8^,^9^,^35^,^36^. Consistent with these observations, we found *Bd* parasitizing zebrafish larvae structures known to express high levels of keratin, such as the edges of the caudal fin^32^, where we observed fin erosion, tissue damage and apoptotic cells. These observations are in agreement with studies showing widespread apoptosis of amphibian skin cells in response to *Bd* infection^6^,^31^. However, in common with many macroparasite disease systems^37^, not all larvae became infected and mortality is heterogeneous among experiments (Supplementary Table 1), suggesting that there are unknown factors underlying the susceptibility of zebrafish larvae to *Bd*.

Heterogeneity in susceptibility of zebrafish larvae to infection may arise from a number of intrinsic and extrinsic factors, investigation of which will likely prove to be fruitful areas of future research using this model. Intrinsic factors include host immune responses and their underlying genetic determinants. Testing the expression of key inflammatory components *caspase-1*, interleukin 1β (*il1β*) and tumour necrosis factor α (*tnfα*) on *Bd*-infected zebrafish larvae cDNA showed no significant difference from controls (Supplementary Fig. 5a). This result is consistent with data from other studies highlighting a complex, immunosuppressive effect on the host immune system that is possibly caused by proteases released by zoospores on infection and by maturing sporangia within the infected epidermis^6^,^38^. As innate immune mechanisms are thought to be highly conserved from zebrafish to man^25^,^39^, the zebrafish response to *Bd* infection is likely to be similar to their amphibian counterparts. The variable expression of inflammatory markers in infected zebrafish may explain some of the heterogeneity that underlies the intensity of parasitism and mortality across our experiments. Owing to destructive sampling of whole larvae for either pathogen DNA or zebrafish RNA, we could not determine associations between the intensity of infection and expression of immunity markers. A future refinement of the model should include the development of an RNA-based marker for *Bd* so that concurrent associations between the intensity of parasitism and onset or type of immunity can be determined. Moreover, the natural translucency of zebrafish larvae enables non-invasive *in vivo* imaging of individual cells and *Bd*-leukocyte interactions at high resolution throughout the organism^22^,^24^,^40^. Strikingly, the major pathogenic events that lead to chytridiomycosis in amphibians^29^, such as hyperplasia of epithelial cells, apoptosis of skin cells and sloughing of infected cells are faithfully reproduced in our zebrafish model. Exploiting this, we have for the first time the ability to examine the biogenesis, architecture, coordination and resolution of the innate immune response to *Bd in vivo*.

Toxins have emerged as critical molecular determinants of tissue damage during fungal infection^5^. We observe that larvae infected with live *Bd* show a greater extent of muscle degeneration when compared to larvae treated with *Bd* zoospore supernatant, and we propose that toxins secreted after sporangia have established in larvae skin play a role in this phenotype. Indeed, studies have shown that *Bd* zoosporangia (in comparison to zoospores) show higher expression levels of genes involved in metabolism and pathogenicity, for example, the carbohydrate-binding module family 18 (CBM18), M36 metalloproteases and crinkler-like virulence effectors^41^,^42^,^43^,^44^. Using the breadth of fluorescent probes available for zebrafish larvae, our *Bd* infection model will greatly enable the *in vivo* investigation of known and unknown pathogenicity factors.

Extrinsic factors, including the virulence of the zoospore suspension, can influence the heterogeneity of infection phenotypes including mortality. Other factors that can influence the outcome of the host-pathogen interactions include the zebrafish microbiome and free-living aquatic predators that prey on infectious *Bd* zoospores^45^. Bacterial flora on amphibian skin has been shown to influence the outcome of *Bd* infection, and probiotic methods to control infection are being widely explored^34^,^46^,^47^. Future work should profile the zebrafish microbiome to better understand its role in governing the process of *Bd*-zebrafish infection, and manipulate the larvae microbiome to dissect the role of component bacterial species in determining the probability of *Bd* infection. To explore these issues, the use of gnotobiotic zebrafish larvae would provide a refinement to the model that has been described here^48^. CFW labelling showed the presence of rare punctae in unexposed control fish; these punctae may signify the presence of fish-associated oomycetes (Saprolegniales) or aquatic fungi other than *Bd*. The addition of low concentrations of antibiotics to zebrafish larvae water during infection is unlikely to remove the natural fungal fauna (the mycobiome) of the larvae in our infection model. Whether or not these commensal eukaryotes have a role in determining infection by *Bd* remains to be investigated. Finally, free-living rotifers are often found inhabiting zebrafish colonies before larvae are washed. Research has shown that aquatic microfauna can predate on the free-living infectious zoospores of *Bd*, thereby lessening the probability of establishing an infection in amphibians^45^. The ability to co-culture rotifers with zebrafish larvae presents an opportunity to further explore the potential manipulation of aquatic microfauna as a method of increasing the resilience of ecosystems to colonization by *Bd.*

Mathematical epidemiological models have shown that the highly virulent nature of *Bd* and its on-going persistence in ecosystems following host extirpations is a function of this parasite's ability to reduce the severity of density-dependent regulation through saprobic growth or parasitism of alternative, more tolerant, hosts^2^,^35^,^49^,^50^. Our finding that *Bd* can infect and proliferate on zebrafish larvae provides evidence that larval fish may represent an alternative reservoir of infection in nature. We tested the extent to which fish are an alternative zoonotic reservoir by infecting a second species, ornamental guppies, which represent a more mature developmental stage than 3 d.p.f. zebrafish larvae. Guppies treated with live *Bd* showed significantly increased GE of *Bd* at 5 d.p.i. compared to those treated with heat killed zoospores, however this signal was lost at 12 and 19 d.p.i. suggesting that infection is unable to persist in this host (Supplementary Fig. 1e). The increasing number of studies that have now shown conflicting evidence for the importance (or otherwise) of various non-amphibian hosts in acting as reservoirs of *Bd* is intriguing^8^,^9^,^10^,^11^. Therefore, the challenge that lies ahead is to more closely examine the biotic and abiotic factors that govern the ability of *Bd* to parasitize alternative hosts, and to understand their contribution to the dynamic nature of chytridiomycosis across various ecological settings. Contributing to this end, we here show that zebrafish larvae represent a tractable and powerful model aquatic system within which to explore the epidemiology and biology of this destructive chytrid.

## Methods

### Ethics statement

Animal experiments conducted at Imperial College or Cardiff University were performed according to the Animals (Scientific Procedures) Act 1986, and were approved by the Home Office (Project Licenses: PPL 707446 or 302876, respectively).

### Zebrafish care and maintenance

Wild-type AB stock was purchased from the Zebrafish International Resource Center (Eugene, OR). Eggs were obtained by placing breeding boats at the base of adult zebrafish tanks overnight, then bleached according to protocols described in The Zebrafish Book^51^. From 0 d.p.f. larvae were kept in petri dishes containing embryo medium (E2) without methylene blue, prepared as described in The Zebrafish Book and washed daily up to and including 3 d.p.f. Larvae were anaesthetized with 400 μg ml^−1^ tricane (Sigma-Aldrich) during *in vivo* imaging.

### Growth of *Batrachochytrium dendrobatidis*

The strain used in this project, JEL423, was first isolated in 2004 from *Phyllomedusa lemur* in Panama and is the isolate from which the Broad Institute *Bd* reference genome was sequenced^52^. To prepare *Bd* zoospore suspension, mTGhL agar plates (8 g typtone, 2 g gelatin hydrosylate, 4 g lactose and 10 g agar to 1,000 ml of water) were seeded with actively growing *Bd* from mTGhL broth culture (0.5 ml). These plates were sealed and incubated for 4–7 days at 17 °C. Zoospores were collected by flooding plates with autoclaved MilliQ (MQ) water (1 ml), rocked back and forth to dislodge zoospores, and left to stand for 10–15 min before collecting the supernatant; plates were flooded twice when necessary. A similar process was repeated with uninoculated control plates to obtain mTGhL plate washings (control). Zoospore concentration was determined by counting zoospores using a haemocytometer. Heat-killed *Bd* zoospores were obtained by heating zoospore suspension at 60 °C for 10 min (zebrafish experiments) or 90 °C for 15 min (guppy experiments). *Bd* zoospore supernatant was obtained by filtering *Bd* zoospore suspension through a 0.45 μm filter to remove all zoospores. All infections were performed within 2 h of collecting zoospores to ensure survival of zoospores when inoculated into fish bath water^36^.

### Zebrafish larvae infections

At 3 d.p.f. individual wild-type AB zebrafish larvae were transferred into 24-well culture plates and infected with *Bd* zoospores (100 μl) in E2 without methylene blue (1 ml), containing 1% penicillin/streptomycin prepared no more than 5 h before infection. Penicillin/streptomycin is commonly used to isolate *Bd* from infected amphibians^53^. Larvae infected with *Bd* were then incubated at 21 °C. All infection experiments were incubated with the initial inoculum of *Bd,* with no changes of water except in experiments conducted in Fig. 1a and Supplementary Fig. 1a where water was changed at 24 h.p.i. All experiments were terminated at 72 h.p.i. unless stated otherwise. Doses are categorized as follows, low dose=final *Bd* zoospore concentration<200 zsp per μl; high dose=final *Bd* zoospore concentration>200 zsp per μl. Control larvae represent larvae treated with 100 μl of mTGhL plate washings. Un-inoculated larvae represent larvae incubated in E2 only for the entire duration of the experiment.

### DNA extraction and *Bd* qPCR

Genomic DNA from zebrafish swabs was extracted using the bead-beating protocol published by Boyle *et al*.^26^ and adapted for zebrafish larvae as follows. Anaesthetized larvae at 1, 24, 48, 72 and/or 96 h.p.i. together with 10 μl of their infected bath water were transferred into 1.5 ml screw top centrifuge tubes containing 50 μl of PrepMan Ultra (Applied Biosystems) and 30–40 mg of 0.5 mm silica beads. Note that in time-course experiments, samples containing larvae and infected bath water were frozen at −20 °C until 96 h.p.i., when extraction was performed on all samples. Samples from dose dependent studies were also extracted in parallel. Larvae were homogenized for 60 s in a mini beadbeater, and then centrifuged for 30 s at 1.3 × 10^4^ r.p.m. in a microfuge. Homogenization and centrifugation was repeated twice. Samples were heated to 100 °C in a heat block, cooled for 2 min, then centrifuged for 3 min at 1.3 × 10^4^ r.p.m. before collection of the supernatant. This process was repeated with 10 μl of infected bath water alone as a control for each sample, and with control treated larvae and bath water as a control for the experiment. A 1/10 dilution in autoclaved MQ water of all supernatants were made before freezing extracted samples and dilutions at −20 °C, kept for a maximum of 48 h post extraction.

Guppy DNA was extracted from samples suspended in 100% ethanol using DNeasy Blood and Tissue Kit (Qiagen), following the manufacturer's protocol. Extracted DNA samples were used without dilution and kept at −20 °C.

Real-time Taqman-qPCR assays to amplify *Bd* DNA were performed with Applied Biosystems 7300 using 25 μl reactions containing 2xTaqman Master Mix (Applied Biosystems), primers 5.8S (5′-AGCCAAGAGATCCGTTGTCAAA-3′) and ITS-1 (5′-CCTTGATATAATACAGTGTGCCATATGTC-3′), TaqMan MGB probe (6FAM 5′-CGAGTCGAACAAAAT-3′ MGBNFQ)^26^. Bovine serum albumin (BSA) at 400 ng μl^−1^ was included to increase the yield of the qPCR. Quantifications were performed on duplicate wells. Each 96-well assay plate included standard reactions containing *Bd* DNA at 1,000, 100, 10 and 1 genomic equivalents (GE) as well as negative controls containing no DNA template. Samples with greater than 0.1 GE were considered positive for *Bd*.

To eliminate background upregulation due to residual *Bd* DNA in zebrafish larvae bath water, data showing GE of *Bd* on zebrafish was normalized using the following formula for all qPCR figures (except in Fig. 1a and Supplementary Fig. 1b where bath water was changed at 24 h.p.i.): GE of *Bd* on zebrafish=(larvae homogenized with 10 μl of its infected bath water)−(10 μl infected bath water alone).

### Histologic sections

Zebrafish larvae at 72 h.p.i. were fixed with 4% paraformaldehyde overnight at 4 °C. Larvae were washed three times in PBS and mounted in 1% agarose. The agarose was dehydrated in a series of ethanol from 70 to 100% and then in 100% xylene and embedded in paraffin. Longitudinal sections of tail were stained with haematoxylin and eosin. Haematoxylin and eosin-stained tissues were imaged with an Axio Lab.A1 microscope (Carl Zeiss MicroImaging GmbH, Germany), and images acquired using an Axio Cam ERc5s colour camera and computer processed using AxioVision (Carl Zeiss MicroImaging GmbH, Germany).

### Microscopy of zebrafish

*In vivo* imaging of *Bd* was performed by staining infected zebrafish larvae with Fluorescent Brightener 28 (F3543, Sigma), also known as calcofluor white (CFW). CFW stains chitin and cellulose and is commonly used to detect *Bd* and other fungal pathogens. Larvae were anaesthetized at 24, 48 and/or 72 h.p.i. with 400 μg ml^−1^ tricaine and stained with 250 μg ml^−1^ CFW prepared in autoclaved MQ water, for 20 min at 21 °C in the dark, only larvae that were alive at the time of microscopy were analysed. To label apoptotic cells in *Bd*-infected larvae, TUNEL staining was performed on anaesthetized 72 h.p.i. larvae using in situ Cell Death Detection Kit (Roche) following the manufacturer's protocol. Images of whole larvae were taken using a Leica M205FA fluorescence stereomicroscope, Leica Macrofluor Z16 APOA (zoom 16:1) equipped with a Leica PlanApo 2.0 × lens, and a Photometric CoolSNAP HQ2 Camera.

For high resolution confocal live imaging of *Bd*, infected larvae were stained at 72 h.p.i. with 1 mg ml^−1^ CFW containing Evans Blue (Sigma) for 10 min at 21 °C in the dark. Evans Blue is a stain used to detect myofibre tissue damage^54^. Larvae were positioned in 35 mm glass bottom dishes (MatTek) and covered with 1% low-melting-point agarose. The immobilized larvae were subsequently covered with E2 (2 ml) containing tricaine.

For antibody labelling, anaesthetized zebrafish larvae were fixed overnight at 4 °C in 4% paraformaldehyde, washed for 3 × 5 min in PBS+0.4% triton, then washed for 1 × 20 min in PBS+1% triton to permeabilize larvae. Anti-*Bd* mAb 5C4 tissue culture supernatant+0.1% sodium Azide (primary antibody) was applied to larvae overnight at 4 °C. Larvae were washed for 4 × 15 min in PBS+0.1% Tween. GFP anti-mouse (to label for *Bd*) and phalloidin (to label for filamentous actin) were diluted 1/200 in PBS and applied to larvae overnight at 4 °C. Larvae were washed for 4 × 15 min in PBS +0.1% Tween. Nuclei were stained with Hoechst diluted 1/500 in PBS, and applied to larvae for 10 min. Larvae were then washed again for 4 × 15 min in PBS +0.1% Tween. Fluorescently labelled larvae were cleared by progressive transfer to 80% glycerol. Larvae were positioned in 35 mm glass bottom dishes and imaged using a Zeiss LSM 710 confocal microscope.

Images were processed using ImageJ software version 10.2 (ref. 55). To measure fin length, images of individual larva were each spatially calibrated using ‘set scale' function (Fig. 2a; Supplementary Fig. 2c). CFW-labelled punctae and TUNEL positive apoptotic cells were also counted using this software (Figs 2d and 3b). To categorize severity of muscle degeneration, larvae were ranked by two observers based on the number of phenotypes larvae possessed when labelled with phalloidin (as described in Supplementary Fig. 3e), none=0, mild=up to 2 and severe=3 or more (Fig. 3e).

### Electron microscopy

For ultrastructure analyses zebrafish larvae at 72 h.p.i. were fixed in 0.5% glutaraldehyde in 200 nM sodium cacodylate buffer for 2 h, washed in buffer and secondarily fixed in reduced 1% osmium tetroxide and 1.5% potassium ferricyanide for 60 min. The samples were washed in distilled water and stained overnight at 4 °C in 0.5% magnesium uranyl acetate, washed in distilled water and dehydrated in graded ethanol, infiltrated with propylene oxide and then graded Epon/PO mixtures until final embedding in full Epon resin in coffin moulds (allowing different orientations) and polymerized at 56 °C overnight. Semi-thin survey sections were cut and stained, final ultrathin sections (typically 50–70 nm) and serial sections were collected on Formvar coated slot grids then stained with Reynold's lead citrate and examined in a FEI Tecnai electron microscope with CCD camera image acquisition.

### Guppy (*Poecilia reticulata*) infections

Adult ornamental guppies (*Poecilia reticulata*) purchased from a commercial supplier were maintained in glass aquaria with suitable refugia containing 50 l of dechlorinated water at 26 (±0.5) °C and 12 h light:12 h dark cycle. Fish were kept in mixed sex (1 male: 16 females) stocks and fed *ad libitum* commercial tropical fish flakes and freshly hatched *Artemia naupli*. Fry were collected twice daily from breeding tanks and transferred to plastic jars containing 800 ml dechlorinated water. Fry were maintained in shoals of five per jar under the same temperature and lighting conditions in a temperature-controlled incubator until the start of the experiment. Seven days before first inoculation, guppies were acclimated to 16 °C over 24 h. These juveniles were aged between 14 and 21 days at time of first inoculation, fed daily and maintained at 16 °C for the duration of experiment. Each group of fish was transferred to a glass crystalizing dish containing 50 ml of dechlorinated water and either 3 × 10^6^ live or heat-killed *Bd* zoospores. After 3 h, guppies and zoospore solutions were gently poured into plastic jars containing 750 ml of dechlorinated water. After 24 h, water was completely changed in each jar. The inoculation process was repeated 48 and 96 h after the initial dose. Guppies were monitored for a total of 19 days. At 5, 9, and 19 days post inoculation 24–33 guppies (17–23 live-*Bd* exposed, 7–10 heat-killed exposed) were killed and preserved in 100% ethanol for subsequent qPCR analysis.

### RNA extraction and host real-time qPCR

Total RNA was extracted from three anaesthetized larvae per sample at 72 h.p.i. using RNAqueous-Micro Total RNA Isolation Kit (Ambion), following the manufacturer's protocol, cDNA was obtained using QuantiTect Reverse Transcription Kit (Qiagen) from 1 μg of RNA per reaction. Quantitative PCR was then performed on a Rotor-GeneQ (Qiagen) using SYBR Green PCR Master Mix (Applied Biosystems). Primers used include *caspase-1* (FW-5′-CTCCATGCAGCCAGCAATTT-3′ and RV-5′-GCAAGGCCAGTCGTTTTCTG-3′), *il1β* and *tnfα* from Stockhammer *et al*.^56^ Quantifications were performed on duplicate wells. To normalize cDNA amounts, we used the housekeeping gene *gadph* (FW-5′-TGGGCCAATGAAGGGAATTCTGGGAT-3′ and RV-5′-TAACAGGTCAGCAACACGATGGCT-3′) and analysed results via the 2^−ΔΔCT^ method^57^. For example, in the case of *tnfα*, 2^−ΔΔCT^ is calculated in three steps: (1) change in cycle threshold (CT) value of *tnfα* compared to housekeeping gene (ΔCT_*tnfα*_)=CT_*tnfα*_ of a sample−CT_*gadph*_ of the same sample; (2) change in CT value of control against infected samples (ΔΔCT_*tnfα*_)=ΔCT_*tnfα*_ of controls−ΔCT_*tnfα*_ of an infected sample; (3) calculate 2^ΔΔCT^_*tnfα*_ for each sample. These calculations were similarly performed for other cytokines and plotted in Supplementary Fig. 5a. Fold induction from all infected samples were also normalized against values from un-inoculated larvae from the same experiment.

### Statistics

These data were statistically analysed with using GraphPad Prism version 7.00 for Mac OS X, GraphPad Software, La Jolla, California, USA, www.graphpad.com (ref. 58). All data were expressed as mean±s.e.m. Significance testing performed by log-rank (Mantel–Cox) test (Fig. 1d), unpaired Student's *t*-test (two tailed) (Figs 1a,b and 2a; Supplementary Figs 1c,e,2c and 5a) and Mann–Whitney test (two tailed; Figs 2d and 3b; Supplementary Fig. 2e).

### Data availability

The data that support the findings of this study are available from the corresponding authors on reasonable request.

## Additional information

**How to cite this article:** Liew, N. *et al*. Chytrid fungus infection in zebrafish demonstrates that the pathogen can parasitize non-amphibian vertebrate hosts. *Nat. Commun.* **8,** 15048 doi: 10.1038/ncomms15048 (2017).

**Publisher's note:** Springer Nature remains neutral with regard to jurisdictional claims in published maps and institutional affiliations.

## Supplementary Material

Supplementary Information Supplementary Figures and Supplementary Table
