# Supplementary material for: Chytrid fungus infection in zebrafish demonstrates that the pathogen can parasitize non-amphibian vertebrate hosts
Source: Nat Commun. 2017 Apr 20;8:15048. doi: 10.1038/ncomms15048 (PMC5411484; doi:10.1038/ncomms15048)
Supplement: Supplementary Information — Supplementary Figures and Supplementary Table [file ncomms15048-s1.pdf]

Supplementary Figure 1. *Bd* infection of zebrafish larvae (Related to Figure 1).

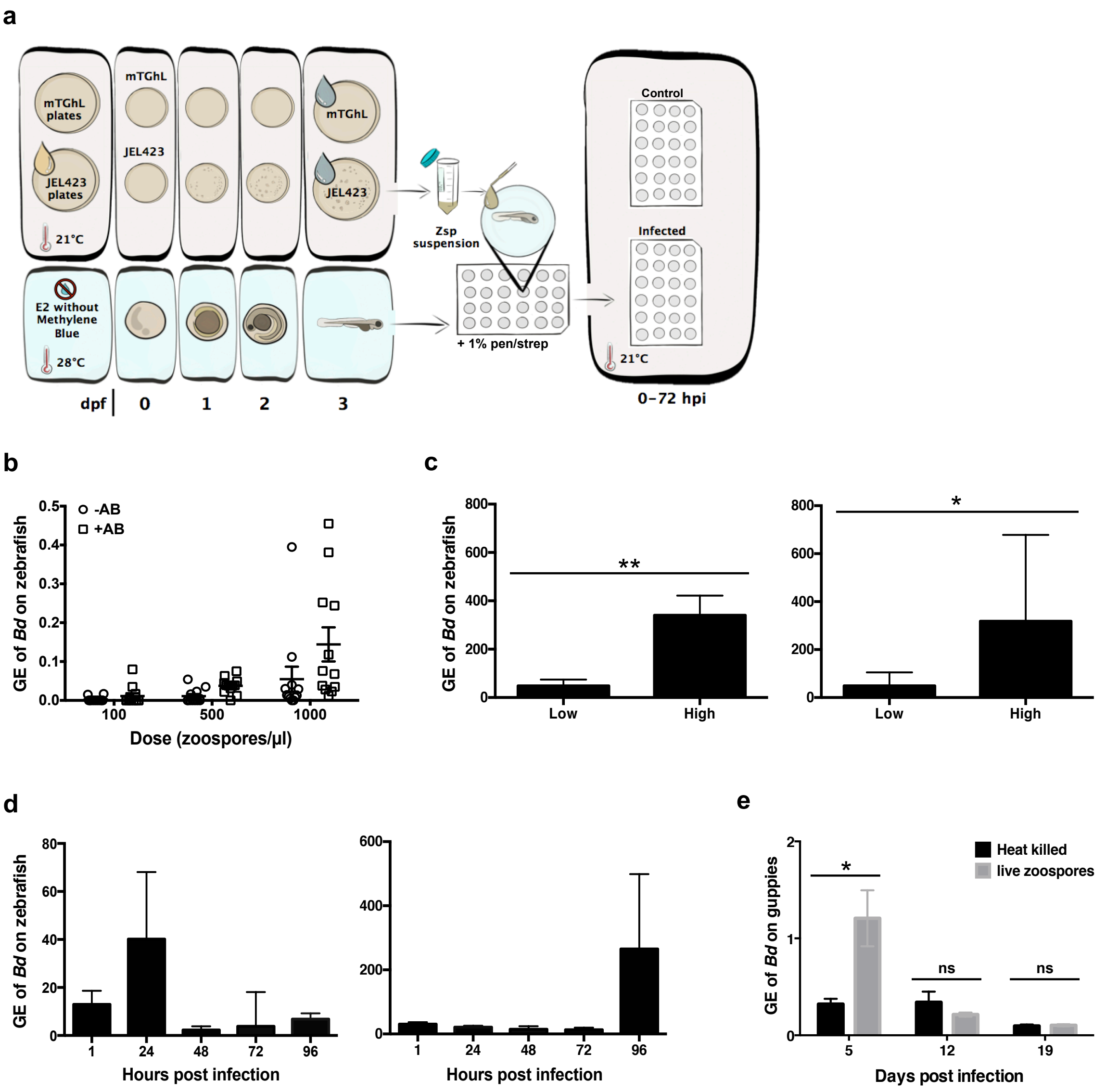

Supplementary Figure 1. *Bd* infection of zebrafish larvae (related to Figure 1). (a) Diagram showing the *Bd*-zebrafish larvae infection model*.* **(b)** Zebrafish larvae bath water without (-AB, open circles) or with (+AB, open squares) 1% penicillin streptomycin was inoculated with three doses of *Bd* zoospores (zsp). Bath water was changed at 24 hours post infection (hpi) and larvae incubated for 72 hpi. DNA from zebrafish larvae was extracted and amplified by qPCR. Genomic equivalents (GE) of *Bd* DNA from one experiment is

plotted here, using n = 12 per treatment. Mean ± SEM are shown. Showing replicate of experiment in Fig. 1a. **(c)** Zebrafish larvae bath water was inoculated with low (< 200 zoospores/µl) or high (> 200 zsp/µl) dose *Bd* zoospores and incubated for 72 hpi. zebrafish DNA was extracted as in **(b)**; GE of *Bd* DNA is plotted here, using n = 12 per treatment. Mean ± SEM are shown. Showing replicates used in Fig. 1b. Significance testing performed using unpaired student's t-test (two-tailed), * $p < 0.05$, ** $p < 0.01$. **(d)** Zebrafish larvae bath water was inoculated with low (< 200 zsp/µl) dose *Bd* zoospores and incubated for 1, 24, 48, 72 or 96 hpi. Zebrafish DNA was extracted as in **(b)**; GE of *Bd* DNA plotted here (dose from left to right = 120 zsp/µl, 170 zsp/µl), using n = 3 per time-point. Mean ± SEM are shown. Showing replicates of experiment in Fig. 1c. **(e)** Juvenile guppy bath water was inoculated with heat killed or live *Bd* zoospores (60 zsp/µl) and incubated for 5, 12 or 19 dpi. DNA from guppies was extracted and qPCR amplified; GE of *Bd* DNA is plotted here, using n = 7 - 23 per time-point. Mean ± SEM are shown. Significance testing performed using unpaired student's t-test (two-tailed), ns $p > 0.05$, * $p < 0.05$.

Supplementary Figure 2. Symptoms of *Bd* infection in zebrafish larvae (Related to Figure 2).

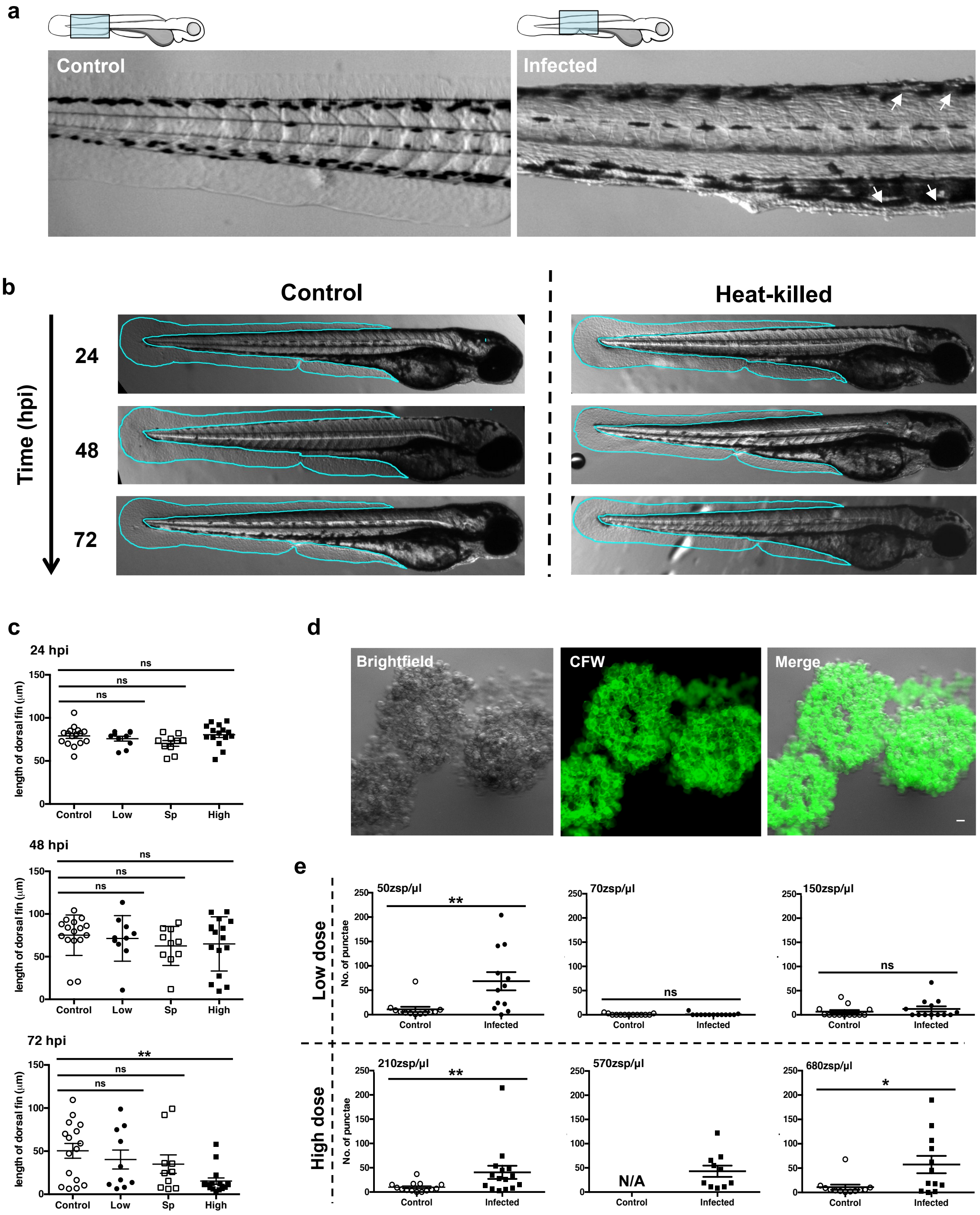

Supplementary Figure 2. Symptoms of *Bd* infection in zebrafish larvae (related to Figure 2). (a) Zebrafish larvae bath water was inoculated with mTGhL (control) or high (> 200 zsp/μl) dose *Bd* zoospores, incubated for 72 hpi and imaged by stereomicroscopy.

Cartoon depicts imaged region. Representative images with arrows highlighting blisters on infected larva. **(b)** Zebrafish larvae bath water was inoculated with heat killed or high dose *Bd* zoospores, incubated for 72 hpi and imaged by stereomicroscopy. Representative images with cyan outline showing presence of fin over time. **(c)** Zebrafish larvae bath water was inoculated with control (open circles), low dose *Bd* zoospores (< 200 zsp/μl, filled circles) high dose *Bd* supernatant (open squares) or high dose *Bd* zoospores (filled squares). Larvae were incubated for 72 hpi and dorsal fin length was measured at 24, 48 and 72 hpi. Each point represents fin length on an individual larva. Data pooled from three experiments per dose, using n = 3 – 4 per treatment. Mean ± SEM are shown. Significance testing performed using unpaired student's t-test (two-tailed), ns $p > 0.05$, *** $p < 0.001$. **(d)** *Bd* broth culture was labelled with calcofluor white (CFW; for chitin, green) using the same protocol as in zebrafish larvae and imaged by fluorescent stereomicroscopy. Representative images showing colocalisation of CFW with *Bd* sporangium. Scale bar = 50 μm. **(e)** Enumeration of CFW-labelled punctae from larvae whose bath water was inoculated with control (open circles), low (filled circles) or high (filled squares) dose *Bd* zoospores and incubated for 72 hpi. Each point represents an individual larva, using n = 12 per treatment. Mean ± SEM are shown. Showing replicate experiments used in Fig. 2d. Significance testing performed using Mann-Whitney test (two-tailed), ns $p > 0.05$, * $p < 0.05$, ** $p < 0.01$.

Supplementary Figure 3. Consequence of *Bd* infection on zebrafish larvae host tissue (Related to Figure 3).

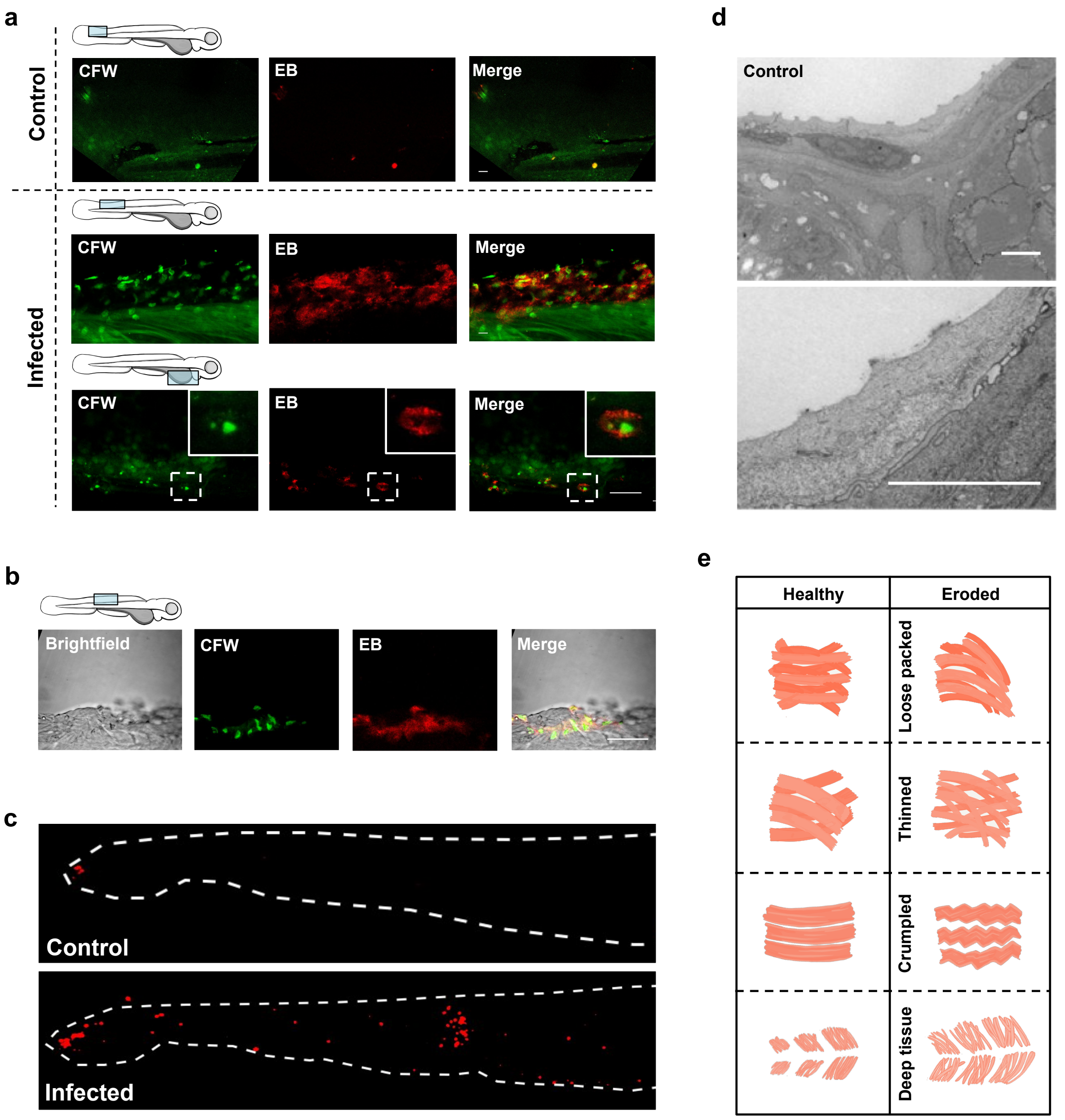

Supplementary Figure 3. Consequence of *Bd* infection on zebrafish larvae host tissue (Related to Figure 3). (a) Zebrafish larvae bath water was inoculated with with mTGhL plate washings (control) or low (< 200 zsp/µl) dose *Bd* zoospores and incubated for 72 hpi, then labelled with calcoluor white (CFW; for chitin, green) and evans blue (EB; for tissue damage, red), and visualised by confocal microscopy. Images taken at 63X, maximum intensity

projection of Z-stack shown here. Cartoon depicts imaged region. Representative images with insets highlight colocalisation of CFW-labelled punctae with EB positive tissue damage. Scale bars = 50 μm. **(b)** Zebrafish larvae bath water was inoculated with high (> 200 zsp/μl) dose *Bd* zoospores, then labelled and imaged as in **(a)**. Scale bar = 50 μm. **(c)** Zebrafish larvae bath water was inoculated with mTGhL (control) or low dose *Bd* zoospores and incubated for 72 hpi, then fixed, labelled with TUNEL (for apoptotic cells; red) and imaged by fluorescent stereomicroscopy. Representative images with dotted outline of larvae tail fin showing an increase in apoptotic cells on infected larvae. **(d)** Zebrafish larvae bath water was inoculated with control and incubated for 72 hpi, then fixed for electron microscopy (EM). Images show healthy larvae skin. **(e)** Diagram showing phenotypes used to categorise severity of muscle degeneration in zebrafish larvae. Left column shows cartoons to illustrate the appearance of healthy muscle; right column shows cartoons for eroded muscle, including loose packing, thinning, crumpling and deep tissue degeneration of muscle fibres.

Supplementary Figure 4. Intracellular colonization by *Bd* in zebrafish larvae (Related to Figure 4).

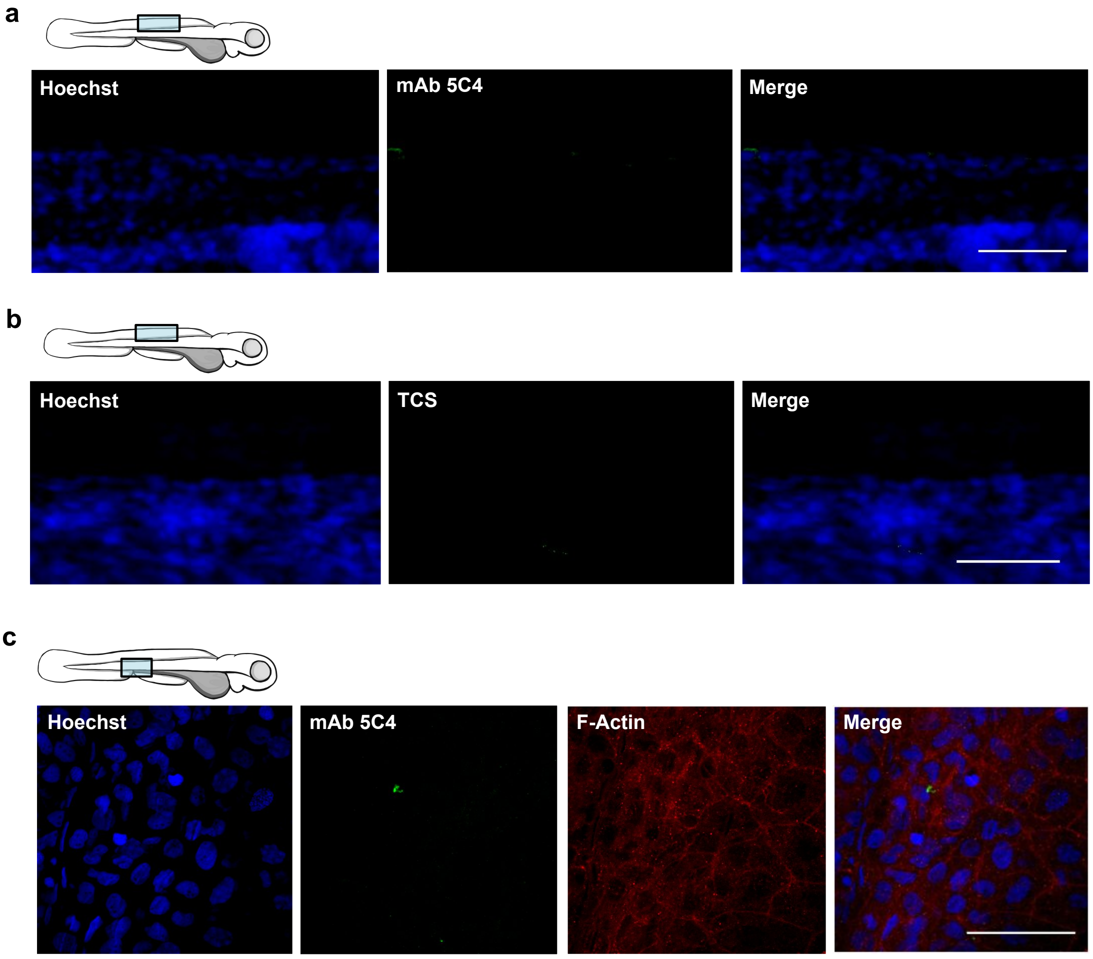

Supplementary Figure 4. Intracellular colonization by *Bd* in zebrafish larvae (Related to Figure 4). (a) Zebrafish larvae bath water was inoculated with mTGhL (control) and incubated for 72 hpi, then fixed and labelled for Hoechst (for DNA; blue) and mAb 5C4 (for *Bd*; green) for visualisation by confocal microscopy. Images taken at 40X, maximum intensity projection of Z-stack shown here. Representative images highlight minimal background labelling of mAb 5C4 in control larvae. Scale bar = 50 µm. **(b)** Zebrafish larvae bath water was inoculated with high (> 200 zsp/µl) dose *Bd* zoospores and incubated for 72 hpi, then fixed and labelled for Hoechst (for DNA; blue) and tissue culture supernatant (TCS;

i.e. mAb 5C4 suspension medium) for visualisation by confocal microscopy. Images taken at 63X, maximum intensity projection of Z-stack shown here. Representative images highlight minimal background labelling of TCS in infected larvae. Scale bar = 50 µm. **(c)** Zebrafish larvae bath water was inoculated with control and incubated for 72 hpi, then fixed and labelled with Hoechst (for DNA; blue), mAb 5C4 (for *Bd*; green) and phalloidin (for F-Actin; red) for visualisation by confocal microscopy. Images taken at 63X, maximum intensity projection of Z-stack shown here. Cartoon depicts imaged region. Representative images show distribution of F-actin in healthy larvae. Scale bar = 50 µm.

Supplementary Figure 5. Testing the expression of key inflammatory components.

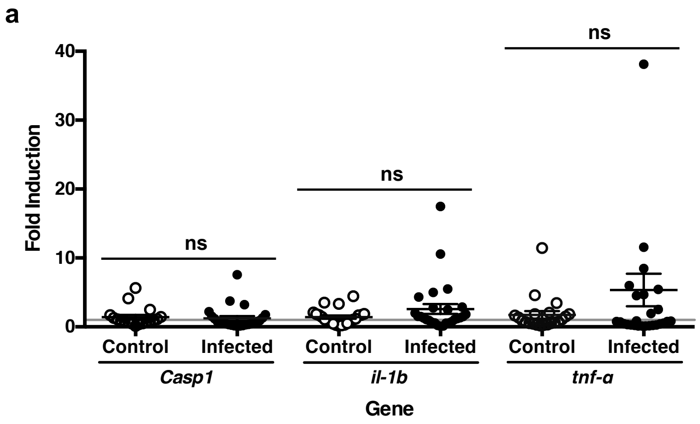

Supplementary Figure 5. Testing the expression of key inflammatory components. (a) Zebrafish larvae bath water was left un-inoculated, inoculated with mTGhL (control) or *Bd* zoospores (19 – 600 zsp/µl) and incubated for 72 hpi. RNA was extracted from pools of 3 larvae per sample. Expression of *caspase-1, il1b, and tnfa* mRNA transcripts were determined by real-time qPCR. Each point shows fold induction from one sample relative to un-inoculated larvae at fold induction = 1 (grey line). Data pooled from 6 experiments, using n = 9 per treatment. Significance testing performed using unpaired student's t-test (two-tailed), ns $p > 0.05$.

Supplementary Table 1. Survival Assays

Date	Zoospore conc. (zsp/µl)	Dose	Sample size (n per treatment)	% Survival					
				24 hpi		48 hpi		72 hpi	
				Control	Infected	Control	Infected	Control	Infected
15/05/2015	170	heat-killed	12	100	100	100	100	100	100
29/05/2015	180	heat-killed	12	100	100	100	100	100	100
02/07/2015	400	heat-killed	12	100	100	100	100	100	100
25/08/2015	120	heat-killed	12	100	100	100	100	100	100
06/10/2014	680	high	12	100	100	100	92	100	67
10/10/2014	40	low	12	100	100	100	100	100	100
10/10/2014	400	high	12	100	100	100	83	100	75
17/10/2014	550	high	12	100	100	100	100	92	100
21/10/2014	700	high	12	100	100	100	100	100	92
27/10/2014	390	high	12	100	100	100	75	100	33
31/10/2014	16	low	12	100	100	100	100	100	100
31/10/2014	160	low	12	100	100	100	100	100	100
07/11/2014	100	low	12	100	100	100	100	100	100
07/11/2014	290	high	24	100	100	100	75	100	62
10/11/2014	192	low	12	100	100	100	100	100	100
11/05/2015	63	low	12	100	100	100	100	100	100
15/05/2015	170	low	12	100	100	100	100	100	100
21/05/2015	280	high	12	100	100	100	100	100	100
28/05/2015	430	high	12	100	100	100	100	100	100
01/06/2015	230	high	12	100	100	100	75	92	67
27/06/2016	800	high	24	100	100	100	63	100	54
03/07/2016	1,010	high	24	100	100	100	67	100	58
08/07/2016	330	high	24	100	100	100	71	83	42
11/07/2016	450	high	24	100	100	100	79	100	58
22/07/2016	410	high	24	100	100	100	71	92	46
25/07/2016	820	high	24	100	100	100	96	88	58
29/07/2016	1,020	high	24	100	100	100	63	100	33
01/08/2016	950	high	24	100	100	100	67	100	33
05/08/2016	1,030	high	24	100	100	100	71	83	63

Supplementary Table 1. Survival Assays. (a) Survival of zebrafish larvae whose bath water was inoculated with mTGhL plate washings (control), heat-killed, low (< 200 zsp/µl) or

high (> 200 zsp/µl) dose *Bd* zoospores, incubated for 72 hours post infection (hpi). Showing replicate experiments used in Fig. 1d.
